# Cocrystal of Ibuprofen–Nicotinamide: Solid-State Characterization and In Vivo Analgesic Activity Evaluation

**DOI:** 10.3390/scipharm86020023

**Published:** 2018-06-04

**Authors:** Yori Yuliandra, Erizal Zaini, Syofyan Syofyan, Wenny Pratiwi, Lidiya Novita Putri, Yuti Sahra Pratiwi, Helmi Arifin

**Affiliations:** 1 Department of Pharmacology & Clinical Pharmacy, Faculty of Pharmacy, Andalas University, Padang 25163, Indonesia; yoriyuliandra@phar.unand.ac.id (Y.Y.); yutisahrapratiwi@gmail.com (Y.S.P.); helmiarifin@phar.unand.ac.id (H.A.); 2 Department of Pharmaceutics, Faculty of Pharmacy, Andalas University, Padang 25163, Indonesia; syofyan@phar.unand.ac.id (S.S.); wennypratiwi0411@gmail.com (W.P.); putrilidya06@gmail.com (L.N.P.)

**Keywords:** ibuprofen, nicotinamide, cocrystalline phase, solubility, in vivo analgesic activity, writhing test

## Abstract

Ibuprofen is classified as a BCS class II drug which has low solubility and high permeability. We conducted the formation of the cocrystalline phase of ibuprofen with coformer nicotinamide to increase its solubility. The purpose of this study was to characterize the solid state of cocrystalline phase of ibuprofen-nicotinamide, determine the solubility, and evaluate its in vivo analgesic activity. The cocrystal of ibuprofen-nicotinamide was prepared by a slow evaporation method. The solid-state characterization was conducted by powder X-ray diffraction (PXRD) analysis, differential thermal analysis (DTA), and scanning electron microscopy (SEM). To investigate the in vivo analgesic activity, 28 male Swiss-Webster mice were injected with acetic acid 0.5% following oral administration of intact ibuprofen, physical mixture, and its cocrystalline phase with nicotinamide (equivalent to 26 mg/kg ibuprofen). The number of writhes was counted, and pain inhibition was calculated. All data were analyzed with one-way ANOVA followed by Duncan’s Multiple Range Test (95% confidence interval). The results revealed that a new cocrystalline phase was successfully formed. The solubility testing showed that the cocrystal formation enhanced the solubility significantly as compared with the physical mixture and intact ibuprofen. A significant increase in the analgesic activity of cocrystal ibuprofen-nicotinamide was also confirmed.

## 1. Introduction

**Figure 1:**
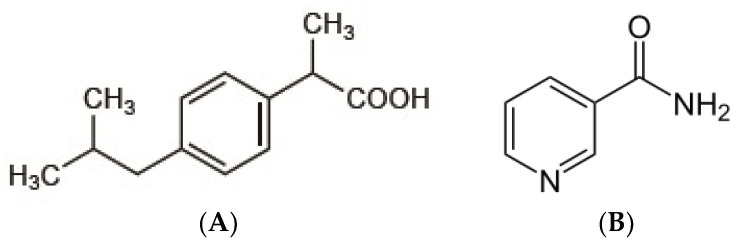
Chemical structures of (**A**) ibuprofen (C_13_H_18_O_2_), and (**B**) nicotinamide (C_6_H_6_N_2_O).

Ibuprofen (Figure 1A) is a potent non-steroidal anti-inflammatory drug mainly used in the treatment of mild to moderate pain and fever. The analgesic activity of ibuprofen has been shown to correlate with its serum concentration. Ibuprofen was classified as a BCS class II drug according to Biopharmaceutical Classification System (low solubility and high permeability). The dissolution in gastrointestinal fluid becomes the rate-limiting step for the absorption process of this drug. Therefore, to reach the quick onset of action, improving the rate of dissolution in the gastrointestinal fluid is necessary [1,2,3].

Some methods have been developed to improve the dissolution rate of ibuprofen, such as preparation of solid dispersion, inclusion complexes, the formation of an amorphous phase, and salt formation [4,5,6]. One of the flourishing techniques in crystal engineering to enhance the solubility and dissolution rate is the formation of co-crystalline phases. The preparation of the cocrystalline phase of ibuprofen with certain coformers has improved the drug’s solubility, intrinsic dissolution rate, and overall performance. The drug shows no likelihood to agglomerate in aqueous media, and can improve its chemical stability [7]. In addition, the mechanical properties and hygroscopicity of the compound are also reported to have improved simultaneously. These also include less sorbed moisture and enhanced tableting behavior [8].

Cocrystals are defined as “solids that are crystalline single phase materials composed of two or more different molecular and/or ionic compounds generally in a stoichiometric ratio, which are neither solvates nor simple salts” [9]. The formation of a cocrystal of active pharmaceutical ingredients (API) with coformers is a promising approach to improving the stability, solubility, and dissolution rate, bioavailability, and mechanical properties of active pharmaceutical ingredients, without any chemical modification [10,11,12,13]. A number of excipients have been approved as “generally recognized as safe” (GRAS) by the FDA as coformers for cocrystal formation. These include nicotinamide, saccharin, citric acid, and tartaric acid [14].

Several previous studies of cocrystal formation of ibuprofen have been reported [15,16,17]. However, the evaluation of in vivo analgesic activity of cocrystal ibuprofen and its comparative effect with intact and physical mixture of ibuprofen has not been reported to our knowledge. The evaluation of the drug effect in experimental animals is necessary to confirm its efficacy in living tissues. In the present investigation, we prepared the cocrystal of ibuprofen (Figure 1A) and coformer nicotinamide (Figure 1B) by solvent evaporation method, and then characterized the solid-state properties by powder X-ray diffraction, thermal analysis DTA, scanning electron microscopy (SEM), and apparent solubility. The in vivo analgesic activity of the cocrystalline phase was carried out by the writhing test method.

## 2. Materials and Methods

### 2.1. Drugs and Chemicals

Ibuprofen and nicotinamide were supplied from Kimia Farma Ltd. (Jakarta, Indonesia). Ethanol, sodium hydroxide, Tween 80, acetic acid 98% *v*/*v*, and sodium chloride were purchased from Bratachem Ltd. (Jakarta, Indonesia). Methanol HPLC grade was purchased from Merck (Darmstadt, Germany). Water used was double-distilled.

### 2.2. Preparation of Physical Mixtures and Cocrystals by Slow Evaporation

A physical mixture of the equimolar ratio between ibuprofen (3.140 g) and nicotinamide (1.8591 g) was prepared manually by slight trituration in a mortar and pestle apparatus for 5 min. This sample was stored in desiccator for further analysis. Meanwhile, cocrystal of ibuprofen-nicotinamide was prepared by a slow evaporation method. An equimolar ratio mixture of ibuprofen (3.140 g) and nicotinamide (1.8591 g) was dissolved in 20 mL ethanol and mechanically stirred until a clear solution was obtained. The solution was kept to allow slow evaporation over 2 days at room temperature. The cocrystallized product was collected and stored in desiccator.

### 2.3. Solid-State Characterization

#### 2.3.1. Powder X-ray Diffraction (PXRD) Analysis

PXRD was conducted by Rigaku RINT-2500 diffractometer (Rigaku Corp., Tokyo, Japan) at 40 kV and 35 mA with Cu Kα radiation source. The samples were scanned from 5° to 35° at a scanning rate 0.5° per minute. The diffractograms were processed using diffraction plotter software (WinPLOTR, Rennes, France).

#### 2.3.2. Differential Thermal Analysis (DTA)

Thermal characterization was carried out using DTG-60 Shimadzu (Kyoto, Japan). Indium was used for calibration. An amount of 3–5 mg samples weighed accurately was deposited in hermetically sealed aluminum pans and scanned from 30° to 220° at a heating rate of 10 °C per minute.

#### 2.3.3. Microscopic Analysis

SEM microphotographs were captured using scanning electron microscope (JEOL type JSM-6360LA, Tokyo, Japan) operated at an excitation voltage of 20 kV and current of 12 mA. The samples were mounted on a double-faced adhesive tape and sputtered with a thin gold-palladium layer.

### 2.4. Solubility Test

The saturated solubility in CO_2_ free distilled water was determined at room temperature using an orbital shaker. Excess amounts of the samples were added to 100 mL of the media, and then filtered through a membrane filter after 24 h of equilibration. The concentration of ibuprofen was determined by the HPLC technique carried out in triplicates. The analysis was performed by using a Shimadzu LC-20AD (Kyoto, Japan) equipped with DAD UV-Vis detector. The HPLC system consisted of a 4.0 × 125 mm LiChrospher RP-18 (Shimadzu, Kyoto, Japan) filled with 5 μm material. A mixture of methanol and water (80:20) was used as the mobile phase. Ibuprofen was detected by UV spectrophotometer at wavelength 266 nm. The retention time (tR) of ibuprofen was 8.22 min.

### 2.5. In Vivo Evaluation of Analgesic Activity

#### 2.5.1. Animal Preparation

A number of 28 male Swiss-Webster mice aged 2–3 months and weighed 20–30 g were used for this study. The animals were kept in standard environmental conditions at room temperature and 12/12-h light-dark cycle for 10 days of acclimation. The experimental protocol was approved by the Ethics Committee of Faculty of Medicine, Andalas University No. 211/KEP/FK/2016 (30 September 2016).

#### 2.5.2. Analgesic Activity Evaluation

The evaluation of analgesic activity was conducted by the writhing method. The mice were divided into control and treatment groups. The treatment groups were subdivided into 3 groups receiving intact ibuprofen, physical mixture of ibuprofen-nicotinamide, and cocrystal of ibuprofen-nicotinamide. All doses were administered by oral gavage at a dose equivalent to 26 mg/kg of ibuprofen. Fifteen minutes following the treatment, each animal received acetic acid 0.5% administered intraperitoneally for pain induction. The writhing movements were observed for 90 min (minute 5, 15, 30, 45, 60, 75 and 90). A writhe was defined as a stretching of the hind limbs accompanied by a contraction of the abdominal muscles. Numbers of writhes were compared to the control group and calculated to obtain pain inhibition percentage by the following equation [18]:

% Inhibition = {(Wc − Wt) × 100}/Wc(1)

where,

Wc = Number of writhes in control group
Wt = Number of writhes in test group

#### 2.5.3. Statistical Analysis

All data from the experiment were presented as mean ± SEM. Statistical analysis was performed by using one-way ANOVA followed by Duncan’s Multiple Range Test. The significance level was taken at 95% of confidence interval. All statistical analyses were carried out using SPSS version 19 for Windows.

## 3. Results

### 3.1. Solid State Characterization

#### 3.1.1. Powder X-ray Diffraction (PXRD) Patterns

**Figure 2:**
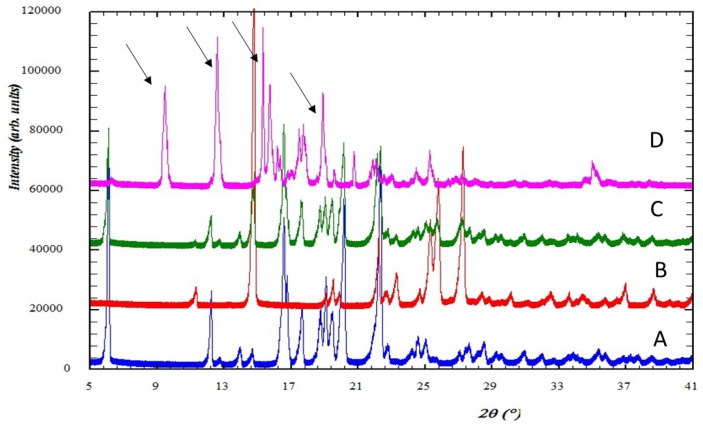
The PXRD patterns for (**A**) intact ibuprofen; (**B**) intact nicotinamide; (**C**) physical mixture of ibuprofen-nicotinamide; and (**D**) cocrystalline phase of ibuprofen-nicotinamide.

The PXRD patterns for intact ibuprofen, physical mixture of ibuprofen-nicotinamide, and the cocrystalline phase are depicted in Figure 2. The diffraction pattern of intact ibuprofen shows that the solid drug is a highly crystalline powder and with sharp diffraction peaks at 2Ɵ equal to 6.00, 12.1, 16.53, 17.48, 20.16, and 22.33. Meanwhile, the characteristic diffraction peaks of intact nicotinamide appear at 2Ɵ equal to 14.74, 22.20, 23.16, 25.26, 25.65, and 27.18. The cocrystal shows the diffraction peaks at the following 2Ɵ angles = 9.51, 12.64, 15.19, 15.57, 17.67, 18.76, 20.80, 21.82, 25.07, and 34.83.

#### 3.1.2. Differential Thermal Analysis (DTA) Thermogram

**Figure 3:**
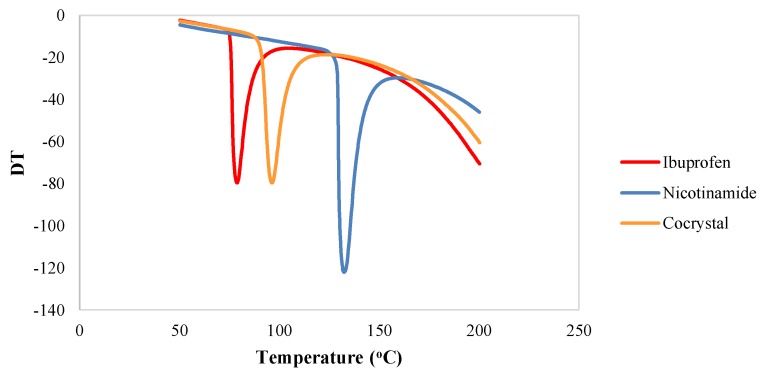
Differential thermal analysis (DTA) thermogram for ibuprofen, nicotinamide, and their cocrystalline phase.

DTA analysis was utilized to study the thermal behavior of the cocrystal of ibuprofen-nicotinamide in relation to the individual components. The DTA thermograms for ibuprofen, nicotinamide, and their cocrystalline phase are presented in Figure 3. Ibuprofen shows a single endothermic peak with Tmax = 79.04 °C and enthalpy (ΔH) = −905.52 J/g. On the other hand, nicotinamide also demonstrates a single endothermic peak attributed to melting transition at 132.35 °C with an enthalpy of (ΔH) = −1.51 KJ/g. Surprisingly, the DTA thermogram for ibuprofen-nicotinamide cocrystal shows a new single endothermic transition attributed to the melting transition of the cocrystalline phase (Tmax = 96.24 °C, ΔH = −1.01 KJ/g).

#### 3.1.3. Scanning Electron Micrographs

**Figure 4:**
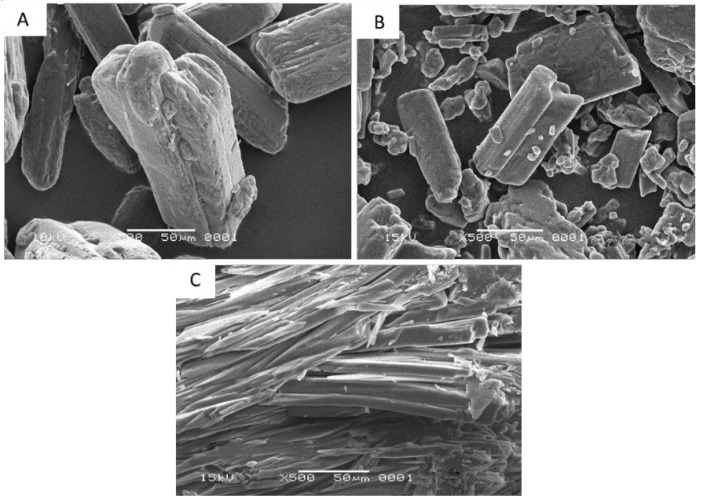
Scanning electron micrographs (**A**) intact ibuprofen; (**B**) intact nicotinamide; and (**C**) cocrystalline phase of ibuprofen-nicotinamide.

The analysis of scanning electron microscopy (SEM) is used to examine the size and crystal habit of a solid phase. The SEM micrographs of intact ibuprofen, intact nicotinamide, and their cocrystalline phase are shown in Figure 4. Crystal habit of intact ibuprofen and intact nicotinamide present as block-shaped particles. On the other hand, their cocrystalline phase is in needle-shaped aggregates.

### 3.2. Solubility Measurement

**Figure 5:**
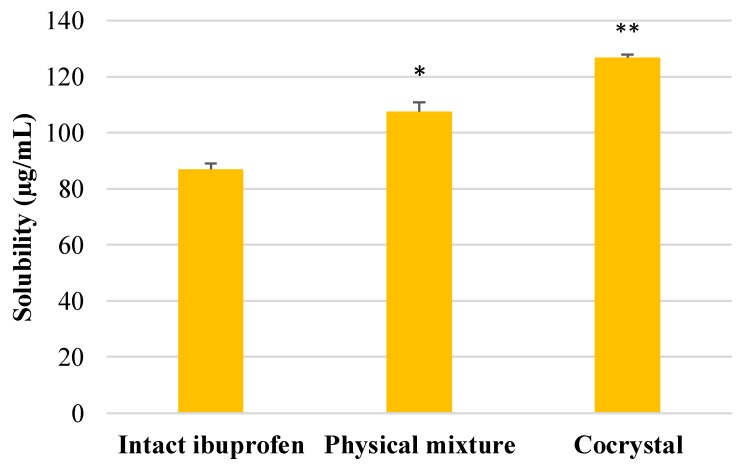
The solubility of intact ibuprofen and its physical mixture and cocrystalline phase with nicotinamide tested in distilled water. * *p* < 0.05 and ** *p* < 0.01 as compared with intact ibuprofen (analyzed with Duncan’s MRT following one-way ANOVA with 95% confidence interval, *n* = 9).

The result of solubility determination of ibuprofen, physical mixture, and cocrystalline phase is presented in Figure 5. This result indicated that there was a significant increase in solubility in both physical mixture and cocrystalline phase of ibuprofen-nicotinamide as compared with intact ibuprofen. Moreover, the cocrystal of ibuprofen also showed a significant improvement in solubility compared with its physical mixture.

### 3.3. In vivo Analgesic Activity of Cocrystal Ibuprofen

**Figure 6:**
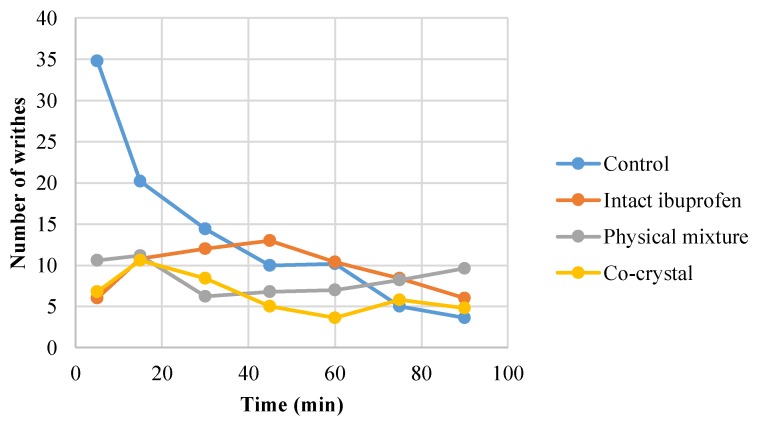
The trend of writhing count of mice induced with acetic acid during 90 min of observation (*n* = 28).

**Figure 7:**
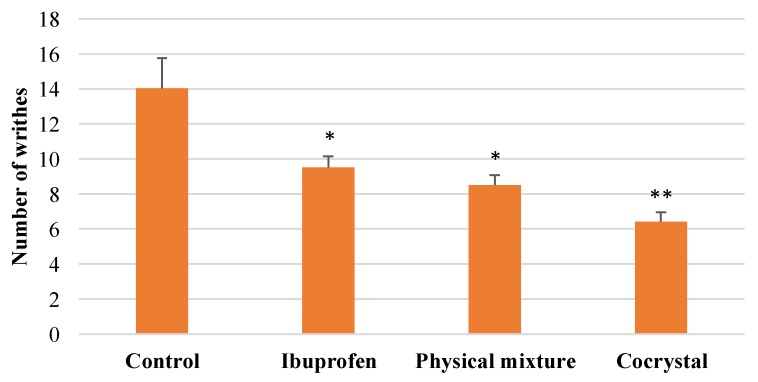
Comparative average number of writhes between groups. * *p* < 0.05 and ** *p* < 0.01 as compared with control group (analyzed with Duncan’s MRT following one-way ANOVA with 95% confidence interval, *n* = 28).

The present study revealed that intact ibuprofen, its physical mixture, and cocrystalline phase with nicotinamide reduced the number of writhes over time during 90 min of observation (Figure 6). The average number of writhes is presented in Figure 7, showing that the cocrystal ibuprofen significantly decreases the number of writhes, as compared with intact ibuprofen and the physical mixture.

The extent of analgesic activity of the drugs are calculated as percent pain inhibition based on the total number of writhes during 90 min of observation. The study showed that the cocrystal exhibited the highest extent of pain inhibition and could attenuate the pain twofold better as compared with intact ibuprofen and the physical mixture of ibuprofen-nicotinamide (Table 1).

## 4. Discussion

The development of pharmaceutical compounds is mostly subjected to the solubility problem, regardless of the solid forms of the compounds. This problem can interfere the bioavailability of the drug; thus, a good molecule of the compound may not make a good drug due to this obstacle [19]. The use of pharmaceutical cocrystal is an emerging approach to be implemented in the pharmaceutical industry. The main purpose of preparing cocrystal is to modify physical properties of drug compounds without altering their pharmacological effects [13]. Ibuprofen is known as a highly permeable analgesic drug with low solubility. Improving the solubility of the molecules is a key factor in achieving the desired effect. The preparation of cocrystal of ibuprofen with nicotinamide by using ethanol as the solvent is expected to enhance its solubility and analgesic activity.

The formation of cocrystal of ibuprofen with nicotinamide has been reported extensively within the last decade. The most significant advantages of this approach is the improved dissolution performance and mechanical properties of the drug [8]. The formation of cocrystalline phase of ibuprofen with other carriers has also been reported. These include 2-aminopyrimidine and other pyridine derivatives [15,20] and levetiracetam [21]. However, nicotinamide is considered as better carrier, as it is categorized as GRAS (generally recognized as safe). Although the cocrystalline phase of ibuprofen with other GRAS carriers had been studied, these reports were only focusing the characterization of the cocrystalline phase. Meanwhile, the present study investigated not only solid-state characteristics and the solubility, but also comparative in vivo evaluation of analgesic activity of the cocrystalline phase of ibuprofen. 

The PXRD analysis showed that the patterns for ibuprofen and nicotinamide in the present study were similar to those reported by Kelly et al. [16]. On the other hand, the physical mixture of ibuprofen and nicotinamide (equimolar) shows the characteristics diffraction pattern of the intact components at similar angles, indicating that no interaction occurs during the mixing process. A unique powder X-ray diffraction pattern for the cocrystal of ibuprofen-nicotinamide, as compared with the patterns of their intact constituents, is strong evidence of the formation of a new cocrystal phase [22]. Although the X-ray diffraction analysis of single crystal to characterize the cocrystalline phase of this system was not conducted in the present study, the purity of the compound can be confirmed by comparing the XRD profile with previous study. Berry et al. has reported the XRD pattern of this system in detail, showing similar interference patterns and peaks [23]. The XRD pattern of the present study also confirmed the finding from a study by Chow et al. [8].

The SEM microphotographs (Figure 4) show that the cocrystalline phase of ibuprofen with nicotinamide are in needle-shaped aggregates, different to its originating compounds that present as block-shaped particles. A different crystal phase of solid particle often demonstrates a different crystal habit [24,25]. The crystal habit may influence the nature of bulk particle such as powder flow properties, bulk density, and compressibility of the cocrystal [26].

The drug compounds in the solid state administered orally must undergo a dissolution process in gastrointestinal fluid before they reach systemic circulation. An adequate amount of drug molecules in target tissues is necessary to obtain rapid and effective pharmacological effects. Hence, the solubility is one of the physicochemical properties that is important in determining the rate of dissolution and the degree of absorption of drug molecules in the gastrointestinal tract [27]. The present study showed that the solubility of the cocrystalline phase of ibuprofen with nicotinamide was significantly better, compared with their physical mixture and intact ibuprofen (Figure 5). The enhancement of ibuprofen solubility in the cocrystalline phase may present through several mechanisms. These include the hydrotropic effect of coformer nicotinamide, and the changes of internal composition of crystal lattice molecules that cause increased affinity in aqueous medium [12,28]. Nonetheless, it has been well recognized that changes of the solid phase of drug compounds, such as amorphous phase, solvate, hydrate, and cocrystalline phase, can improve the solubility and bioavailability of pharmaceutical compounds [29,30,31].

Experimental studies in animals are crucial to follow up in vitro evaluation of drugs. This is because the solubility of a drug is not only a concern of its crystallinity and lipophilicity, but also influenced by the medium into which it will dissolve. Several physiological factors may also interfere with the metabolism of the drugs in the body. These include the transit time of the drug in the gastrointestinal tract prior to reaching its absorptive sites, its residence time in the absorptive sites, the stability of the drug in the luminal fluid, and the possibility of first-pass metabolism [32]. Accordingly, in vitro dissolution study may not be entirely predictive of the actual performance of pharmaceutical dosage forms in the body.

The evaluation of the analgesic effect of ibuprofen in the present study was conducted by the writhing method in mice. This method is one of the most commonly used techniques to evaluate peripherally-acting analgesic activity. The pain was induced by injecting acetic acid solution intraperitoneally. This compound is known to induce systemic pain that alters the cell membranes. Arachidonic acids released by the membranes are converted to prostaglandins that produce the pain by cyclooxygenase enzyme. The increased production of prostaglandin further enhances the vascular permeability. A writhe is counted when the animals are arching their back, extending their hind limbs, and contracting their abdominal musculature. Therefore, decreased number of writhes indicates lower production of prostaglandins [33].

The in vivo evaluation of the analgesic effect of the drugs in this study showed that the three types of ibuprofen were effective at decreasing the number of writhes from the beginning of observations. Although the writhing trend in the animals during 90 min of observation (Figure 6) may not clearly demonstrate the superiority of cocrystal ibuprofen in reducing pain, the comparative average number of writhes between groups (Figure 7) can reveal that the cocrystal of ibuprofen-nicotinamide exhibited the best analgesic effect and was significantly better compared to intact ibuprofen and its physical mixture. Moreover, the pain protection effect of the drugs presented in Table 1 explicitly shows that the cocrystal improved the analgesic effect with almost a twofold increase.

The absorption process of BCS II class drugs such as ibuprofen in the gastrointestinal tract is limited by the low solubility and slow dissolution rate. The low solubility of these drugs may cause inconsistency for a complete absorption, despite the fact that they are highly membrane permeable [34]. Herein, the formation of cocrystalline phase of ibuprofen-nicotinamide could solve these limiting characteristics. The study showed an improved solubility that could simultaneously increase the analgesic effect in the experimental animals.

The cocrystalline phase is a part of multicomponent crystal other than salts, hydrates, and solvates. The crystal engineering of ibuprofen with some pharmaceutical excipients to improve its solubility and analgesic activity has been reported, including through salt formation. The salt of multicomponent crystal of ibuprofen with several excipients such as t-butylamine, tromethamine, and meglumine can significantly improve the solubility of ibuprofen in aqueous media [35,36,37]. The salt of ibuprofen with L-arginine and lysine has also been reported to exhibit better absorption, and increase the onset of action in volunteer patients [38,39].

## 5. Conclusions

The present study reveals that the cocrystalline phase of ibuprofen with nicotinamide exhibits a unique X-ray diffraction pattern that is different to its originating compounds. The thermal behavior of the cocrystal shows a sharp endothermic peak at the temperature of 96.24 °C. The results of the study conclude that the preparation of cocrystalline phase of ibuprofen with nicotinamide significantly enhances the solubility of this poorly soluble drug. The cocrystal also improves the in vivo analgesic effect, as compared with intact ibuprofen and its physical mixture.

## Figures and Tables

**Table 1:** Comparative pain inhibition between groups.

No	Compound	Total Number of Writhes	Pain Inhibition (%)
1	Control	98.2 ± 4.76	0
2	Intact ibuprofen	66.6 ± 2.63	32.18
3	Physical mixture	59.6 ± 2.7	39.31
4	Cocrystalline phase	45.0 ± 5.34	54.17

Data are presented as mean ± SEM, *n* = 28.
